# Supplementary figures and images for: Degradation of chondroitin sulfate A by a PUL-like operon in Tannerella forsythia
Source: PLoS One. 2022 Sep 16;17(9):e0272904. doi: 10.1371/journal.pone.0272904 (PMC9481042; doi:10.1371/journal.pone.0272904)

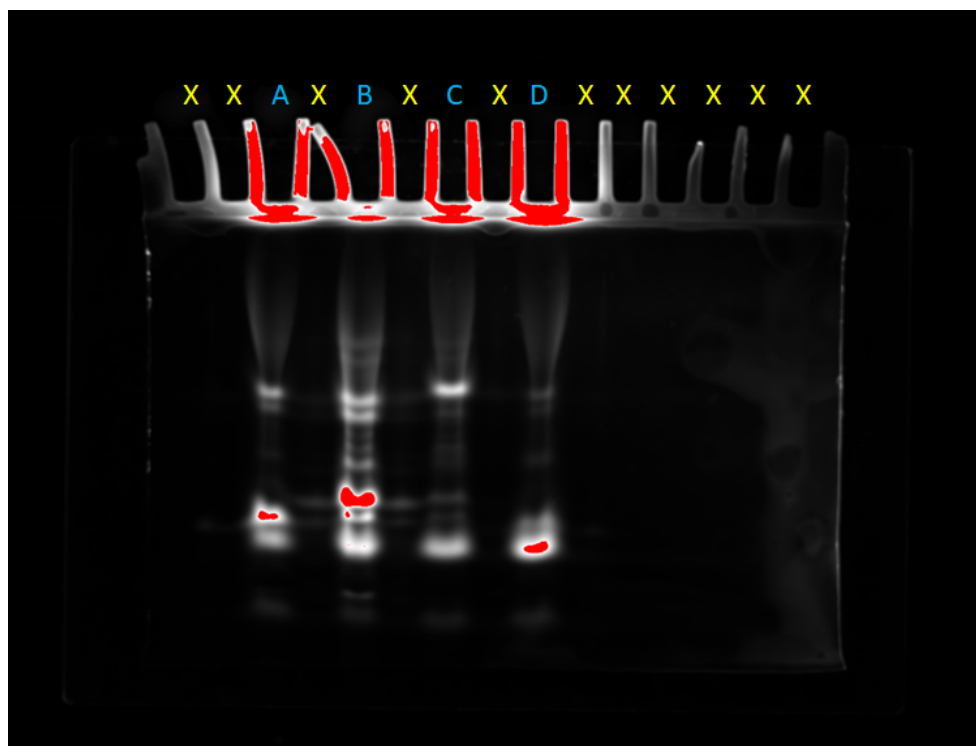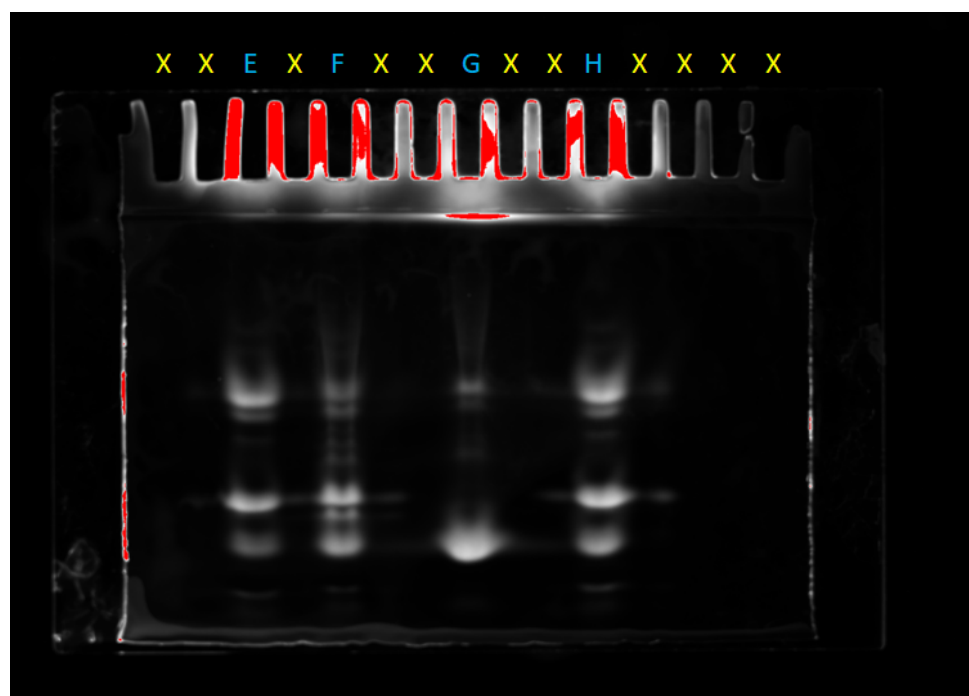

Supplement: S1 Fig — Gel 1 was used in the creation of Fig 3A–3D, and gel 2 was used in the creation of Fig 3E–3H. The gel was loaded from left to right in the annotated order. Experimental samples are labelled using the relevent figure subheading and coloured blue. Blank lanes are labelled X in yellow. Lanes were cropped, rearranged to omit blank lanes, and color inverted using Adobe Photoshop CS6 in order to create the figure. (PDF) [file pone.0272904.s001.pdf]
