# Supplementary material for: Degradation of chondroitin sulfate A by a PUL-like operon in Tannerella forsythia
Source: PLoS One. 2022 Sep 16;17(9):e0272904. doi: 10.1371/journal.pone.0272904 (PMC9481042; doi:10.1371/journal.pone.0272904)
Supplement: S1 Table — Amino acids highlighted in red were removed in the truncation, while amino acids highlighted in green were recombinantly expressed. (DOCX) [file pone.0272904.s002.docx]

Gel 1

**Table S1.** Truncated amino acid sequences of Bfo2285, Bfo2290, and Bfo2291 used for recombinant expression in *E. coli.* Amino acids highlighted in red were removed in the truncation, while amino acids highlighted in green were recombinantly expressed.

Gel 2

| Protein | Amino acid sequence |
| --- | --- |
| Bfo2285 | MKVNVFLISAFLFLVFFGCSPKETTLRNTEICSEEDSVSSVIRTAMQQIGFQVRLIEESGRILNPRTVHNGKVQYISKDDWTSGFFPGTLFYMYDLTGEECWKVTGVKYTEDLDSVKYLKWHHDVGFMINSSFGNALRVTGNEAYKEVLIEAAKSLATRFRPTAGVIQSWDEDRGWQGKRGWMCPVIIDNMMNLELLFKATALSGDSAYYKIAVSHADVTMDNHFREDGSCYHVVDYDKKQGGVRGKYTAQGYADESSWARGQAWAIYGYAVCYRYTRDQRYLEMADRIYRFMFRHKNLPEDLVPYWDYDAPCIPNEPRDASAAAITASALYELASFGKTDYRVTADKIIRSLSSSSYKAIVGTNGNFLLMHSVGSIPHNNEIDVPLNYADYYFLEALIRKKNMK |
| Bfo2290 | MKASKLSCLGLIGVIPACIHAQEHRADDTLRPNIIYIFPDQMRNSAMGFWNDPAFASHLQGKADPVETPNLNRFARESVVFSSAMSNCPLSSPHRASLLTGMYPHRSGVPLNVNSRRPFSTLRNDATTVSDVFSRNGYDCAYIGKYHLDTPTPNDPENPGNYVENRDLVWDAYTPPERRHGFNFWYSYGTFDVHKHPHYWDTDGKRHDINQWSPSHETDMAISYLKNEFGRRDVSKPFFLMISMNPPHHPYNSFNDCMEEDYTHYKDRTLSELLVRHNADTTMEKSSSAAYYFAQITGVDREFGRLLEALDELGLSKNTMVVFSSDHGETMCSHGLQDAKNSPYIESMNVPFLIRYPQRLKPKVVDYLLSSPDIMPTLLGLSNLGQHIPHEVQGTDFSKALFSNQPDKPLPDAALYIRNMDGRQDQDGKVRTYVPVARGIKTHRYTLSLTVDKENKQLKEILLFDDLDDPYQMNNIDWNTRPQLKRQLLIQLGQLLKKYDDPWYKDGILKDLIMYE |
| Bfo2291 | MKKLLFSLIIYFLPVCTLFAYTEKNLLQRKTSEERLKELLVLHQQWVAYPDYTDRQGWDALFGEFKDVYIRRGEKALEYRWQVIQAMDYIEYERSGSRKIMENPYNENAQTLTDLFLAELAEGKGRFIEPLANGVFYFCEMTSWALSAHVRLQHDNRTLPNHGEHVIALVSAEVGAELSWIHYFFRDELDKINPVISQRVRDEVEKRILKTYLETDHFWWMAMNYKPGDMVNNWNPWCNFSVLQCFMLLENDTDRLAKAVYKTIRSVDHFINYSKADGACEEGPSYWGHAAGKLYDYLQLLCDVTGGQLSLFEDPLIRRMGEYISRSYVGNGWVVNFADATARLEPDADLIYRYGKAVNSTEMMAFSAFLRKNEQAPQAPNNRRDVYRTFETLRFHHALSTVTLPYSAPATTWYPETQFCYMRTRGGLFFAGKGGHNNESHNHNDVGTFSLYIDETPVLIDAGVGTYTRQTFGDERYTIWTMQSLFHNLPEINGTQQAFGAKYKAEQMKFTPANRTLSLELKQAYPEETSIHSWNRTYRLRDKQLEINDRFSLESPEKRNKLHFLTWARVDRSIEGQIDMEVQGKKARLSYDRNIFTSSVETITLTDPRLSNVWGKELYRITLEAKELSLSGHYRIVISRK |
