# Supplementary material for: Degradation of chondroitin sulfate A by a PUL-like operon in Tannerella forsythia
Source: PLoS One. 2022 Sep 16;17(9):e0272904. doi: 10.1371/journal.pone.0272904 (PMC9481042; doi:10.1371/journal.pone.0272904)
Supplement: S2 Table — (DOCX) [file pone.0272904.s003.docx]

**Table S2**. Primers for PCR amplification of Bfo2285 from pET21b plasmid for ligation into pET28a.

| **Forward and Reverse Flanking PCR Primers** | |
| --- | --- |
| Forward Flanking Primer | 5’-CGACTCACTATAGGGGAATTGTGAGCGG-3’ |
| Reverse Flanking Primer | 5’-CCGCTCACAATTCCCCTATAGTGAGTCG-3’ |
