# Supplementary material for: Degradation of chondroitin sulfate A by a PUL-like operon in Tannerella forsythia
Source: PLoS One. 2022 Sep 16;17(9):e0272904. doi: 10.1371/journal.pone.0272904 (PMC9481042; doi:10.1371/journal.pone.0272904)
Supplement: Attachment — Submitted filename: Reviewer_Responses.docx [file pone.0272904.s004.docx]

July 22, 2022

Dear PLoS One Publishing and Reviewers,

We are grateful for the work done by the Reviewers and have incorporated the following changes based on their comments.

Reviewer 1 Responses:

- Revised ATKA to ÄKTA.
- 2-aminoacridone changed to 12.5mM from 12.5M

Reviewer 2 Responses:

- Removed words “frequently” and “possible” throughout
- Materials and methods now include protein identities and concentrations used for digestion
- FACE analysis, quantification and standards unavailable. The experiment was conducted to observe 1) how many bands of product were produced by BFO2291 2) how many bands of product were generated from BFO2290 3) BFO2285 active?  Without standards it is inappropriate to assign products to certain bands.  Standards are difficult to obtain for comparison, but we are careful not to draw specific conclusions from this analysis. Attempts at conducting Mass Spec were inconclusive, likely due to the heterogeneity of the source of chondroitin A.

With respect to “Journal Requirements”:

1) We have to the best of our knowledge adhered to the PLOS ONE style requirement
2) We have included the statement "The funders had no role in study design, data collection and analysis, decision to publish, or preparation of the manuscript."
3) The corresponding author, Michael Suits should be affiliated with Wilfrid Laurier University as indicated in the manuscript.
4) The original underlying image for the FACE gel is provided in the supplemental material document.
5) The X-ray diffraction models and datasets presented in this study can be found at the Protein Data Bank: accession numbers 8DI0 and 8DI1 for Bfo2290 and Bfo2294, respectively. The coordinates and structure factors have been communicated to be released immediately.
6) Figure 6 is referred to in the body text.
7) Figure title and caption for the original gel in the supplemental information document follows the other figures in the manuscript.
8) The reference list is complete and correct to the best of our knowledge.

We have no conflicts of interest to disclose. Please address all correspondence concerning this manuscript to [msuits@wlu.ca](mailto:msuits@wlu.ca).

Thank you for your consideration of this manuscript,

**Michael Suits**, Ph.D

Associate Professor

Department of Chemistry & Biochemistry

<http://www.google.com/url?url=http://traces.com/testimonials/&rct=j&frm=1&q=&esrc=s&sa=U&ei=0CGOU4rTE4ypyASvw4EI&ved=0CCQQ9QEwAQ&usg=AFQjCNGPtcHAl63FHI40OFQTeuJxSCXgPQ>

Wilfrid Laurier University

75 University Ave West

Waterloo, ON N2L 3C5

P: 519-884-0710 x 4961

E: [msuits@wlu.ca](mailto:msuits@wlu.ca)
